# Supplementary material for: Regorafenib-Attenuated, Bleomycin-Induced Pulmonary Fibrosis by Inhibiting the TGF-β1 Signaling Pathway
Source: Int J Mol Sci. 2021 Feb 17;22(4):1985. doi: 10.3390/ijms22041985 (PMC7922359; doi:10.3390/ijms22041985)
Supplement: Supplementary file 1 [file ijms-22-01985-s001.pdf]

Supplementary Materials

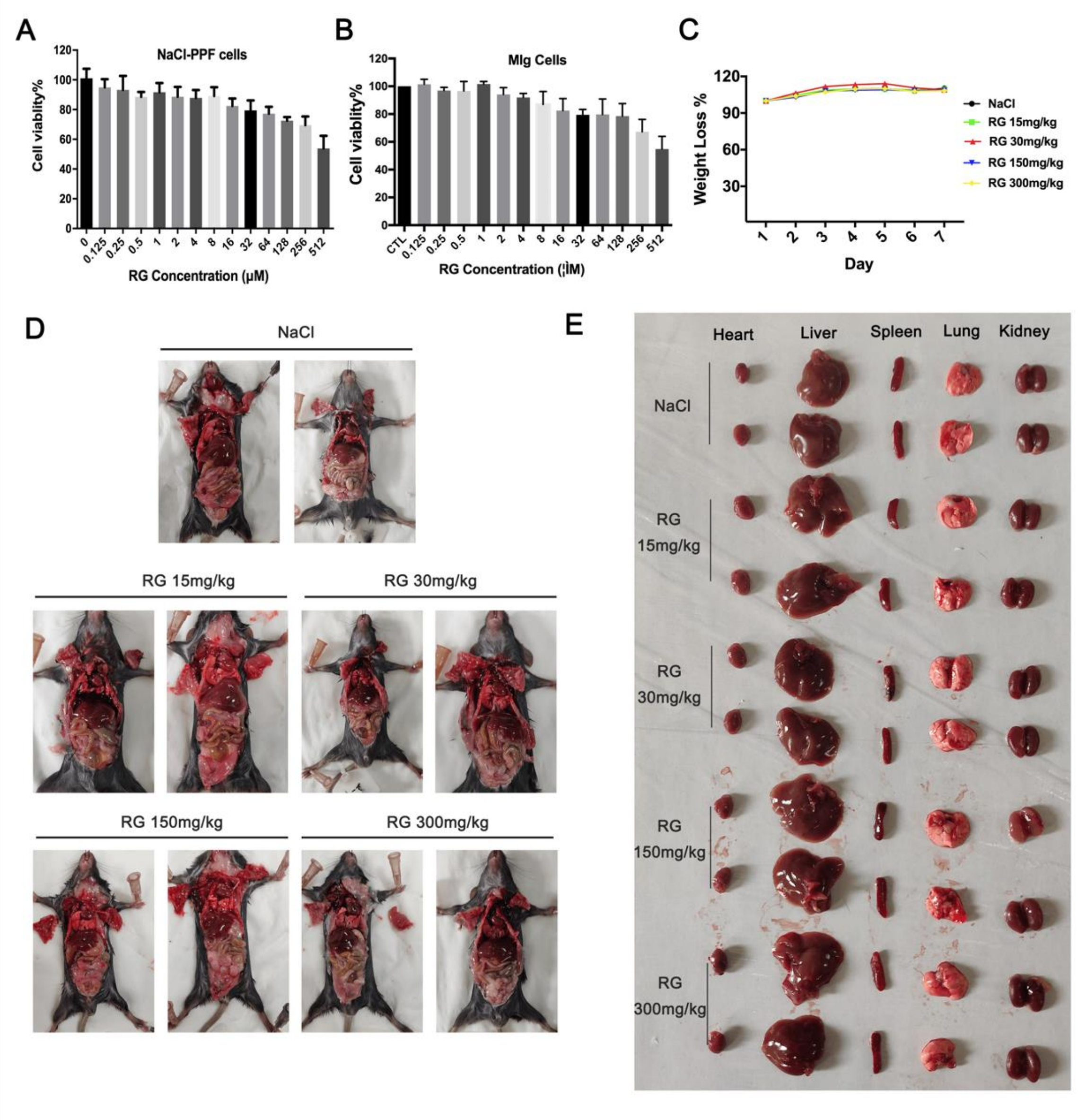

Figure S1. RG has not toxic effect on Mlg and NaCl-PPF cells and C57BL/6j mice. (**A**,**B**) Mlg and NaCl-PPF cells were incubated with RG (0, 0.125, 0.5, 1, 2, 4, 8, 16, 32, 64, 128, 256 and 512 μM) for 24 h, and MTT assays were used to analyze the cytotoxicity effect of RG on the cells; (**C**) RG (15 mg/kg, 30 mg/kg, 150 mg/kg and 300 mg/kg) were given orally once a day for 7 days in normal mice, and the weight changes were shown from day 1 to day 7; (**D**) The appearance of mouse viscera; (**E**) The appearance of five main organs (heart, liver, spleen, lung, kidney).

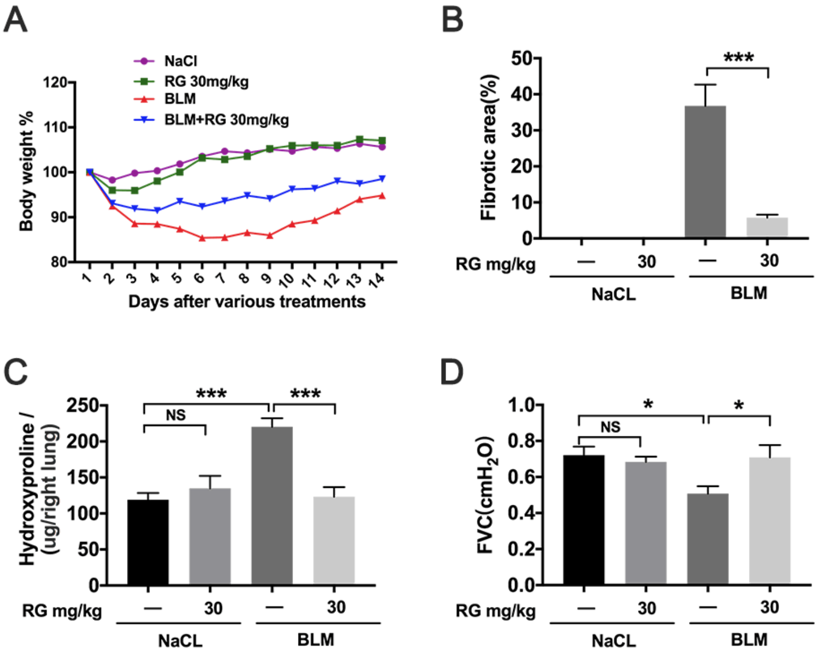

Figure S2. RG attenuates BLM-induced pulmonary fibrosis at a single dose 30 mg/kg. (**A**) RG (30 mg/kg) were given orally once a day from days 7–13 after BLM-treatment, lungs were harvested at day 14, and body weight loss was measured every day; (**B**) Percentages of fibrotic area in lung tissues. (**C**) Hydroxyproline contents in right lung tissues; (**D**) Forced vital capacity (FVC) in each group. Data in (**A**–**D**) are means ± Standard Error of Mean (SEM), * $p < 0.05$, *** $p < 0.001$ (2-way ANOVA), NS: nonsignificant.

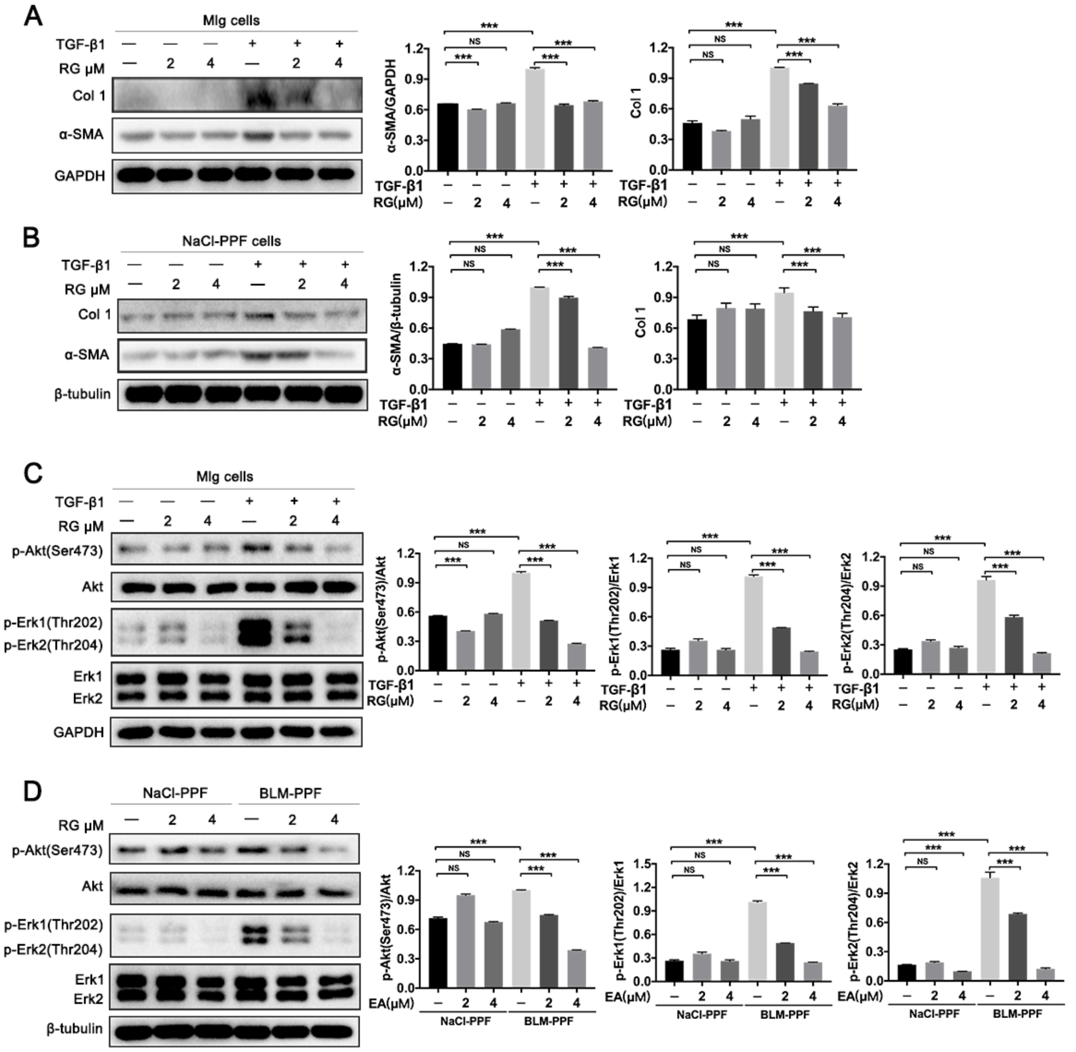

Figure S3. RG suppresses TGF-β1-induced the activation and TGF-β1-non-Smad pathway. (**A**,**B**) Mlg or NaCl-PPF cells were exposed to TGFβ1(5 ng/mL) and/or RG (2 μM, 4 μM) for 24 h to detect the expression levels of α-SMA, Col 1 by Western blot. Densitometric analysis are shown beside; (**C**) Mlg cells were incubated with RG (2 μM, 4 μM) and/or TGF-β1(5 ng/mL) for 12 h to analyze the Erk1/2, Akt and its phosphorylation expression levels by Western blot. Densitometric analysis are shown beside; (**D**) BLM-PPF cells were incubated with RG (2 μM, 4 μM) for 12 h to analyze the Erk1/2, Akt and its phosphorylation expression levels by Western blot. Densitometric analysis are shown beside. β-tubulin or GAPDH were used as a loading control. Data in (**A**–**D**) are means ± Standard Error of Mean (SEM), * $p < 0.05$, ** $p < 0.01$, *** $p < 0.001$ (one-way ANOVA), NS: nonsignificant.

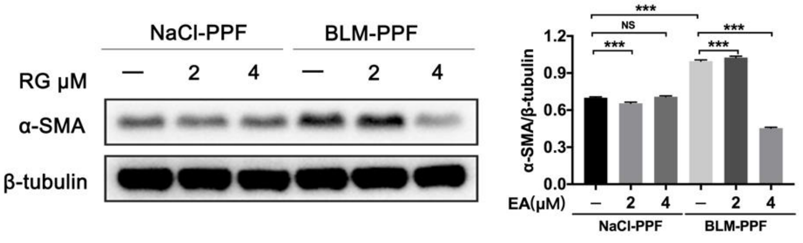

Figure S4. RG inhibits the α-SMA expression level in BLM-PPF cells instead of NaCl-PPF cells. NaCl-PPF and BLM-PPF cells were incubated with RG (2 μM, 4 μM) for 24 h to analyze the α-SMA expression levels by Western blot. Densitometric analysis are shown beside. β-tubulin was used as a loading control. Data are means ± Standard Error of Mean (SEM), * $p < 0.05$, ** $p < 0.01$, *** $p < 0.001$ (one-way ANOVA), NS: nonsignificant.
